# Apollo 3: Multi-Species Genome Curation

**DOI:** 10.64898/2026.06.08.730970

**Published:** 2026-06-11

**Authors:** Garrett J Stevens, Kyösti Sutinen, Dario Beraldi, Shashank Budhanuru Ramaraju, Colin Diesh, William Haese-Hill, Peter Xie, Caroline Bridge, Adrien Morison, Angel Leung, Ulrike Böhme, Scott Cain, Nathan Dunn, Toby Hunt, Jane E Loveland, Adam Frankish, Alexie Papanicolau, Stefano Giorgetti, Jon Keatley, Bethany Flint, Lincoln Stein, Robert Buels, Matt Berriman, Ian Holmes

## Abstract

We present Apollo 3, a new manual genome annotation tool that integrates with the JBrowse 2 genome browser. Its functionality is inspired by existing manual genome annotation tools such as Apollo, Artemis, and Otter, but uses an updated and more scalable architecture and technology stack. It allows the simultaneous editing of multiple genomes, including the visualization of synteny to inform those annotations. Apollo 3 can be used as a standalone annotation editor, or it can be installed on a server and used collaboratively. We describe the application’s design, features, and use cases.

## Full Text Availability

The license terms selected by the author(s) for this preprint version do not permit archiving in PMC. The full text is available from the preprint server.

